# Use of genome-scale models to get new insights into the marine actinomycete genus *Salinispora*

**DOI:** 10.1186/s12918-019-0683-1

**Published:** 2019-01-21

**Authors:** Carolina A. Contador, Vida Rodríguez, Barbara A. Andrews, Juan A. Asenjo

**Affiliations:** 1 Centre for Biotechnology and Bioengineering (CeBiB), Department of Chemical Engineering, Biotechnology and Materials, University of Chile, Beauchef 851, Santiago, Chile; 2 Centre for Soybean Research, State Key Laboratory of Agrobiotechnology, Shatin, Hong Kong; 3 School of Life Sciences, The Chinese University of Hong Kong, Shatin, Hong Kong

**Keywords:** Genome-scale model, Strain-specific adaptation, Metabolic capabilities, *Salinispora* species

## Abstract

**Background:**

There is little published regarding metabolism of *Salinispora* species. In continuation with efforts performed towards this goal, this study is focused on new insights into the metabolism of the three-identified species of *Salinispora* using constraints-based modeling. At present, only one manually curated genome-scale metabolic model (GSM) for *Salinispora tropica* strain CNB-440^T^ has been built despite the role of *Salinispora* strains in drug discovery.

**Results:**

Here, we updated, and expanded the scope of the model of *Salinispora tropica* CNB-440^T^, and GSMs were constructed for two sequenced type strains covering the three-identified species. We also constructed a *Salinispora* core model that contains the genes shared by 93 sequenced strains and a few non-conserved genes associated with essential reactions. The models predicted no auxotrophies for essential amino acids, which was corroborated experimentally using a defined minimal medium (DMM). Experimental observations suggest possible sulfur accumulation. The Core metabolic content shows that the biosynthesis of specialised metabolites is the less conserved subsystem. Sets of reactions were analyzed to explore the differences between the reconstructions. Unique reactions associated to each GSM were mainly due to genome sequence data except for the ST-CNB440 reconstruction. In this case, additional reactions were added from experimental evidence. This reveals that by reaction content the ST-CNB440 model is different from the other species models. The differences identified in reaction content between models gave rise to different functional predictions of essential nutrient usage by each species in DMM. Furthermore, models were used to evaluate in silico single gene knockouts under DMM and complex medium. Cluster analysis of these results shows that ST-CNB440, and SP-CNR114 models are more similar when considering predicted essential genes.

**Conclusions:**

Models were built for each of the three currently identified *Salinispora* species, and a core model representing the conserved metabolic capabilities of *Salinispora* was constructed. Models will allow in silico metabolism studies of *Salinispora* strains, and help researchers to guide and increase the production of specialised metabolites. Also, models can be used as templates to build GSMs models of closely related organisms with high biotechnology potential.

**Electronic supplementary material:**

The online version of this article (10.1186/s12918-019-0683-1) contains supplementary material, which is available to authorized users.

## Background

*Actinomycetes* are a well known source of specialised metabolites including many that possess pharmacological properties such as antibiotics and anticancer agents. The marine actinomycete genus *Salinispora* is no exception [1]. *Salinispora* strains are heterotrophic, obligately aerobic, Gram-positive bacteria belonging to the family *Micromonosporaceae* [2]. Members of the genus have growth requirements for medium with high ionic strength [3]. Currently, the genus encompasses three species that have been validly recognized and characterized: *S. tropica*, *S. arenicola*, and *S. pacifica* [4, 5]. *Salinispora* strains are widely distributed in tropical and subtropical marine environments with a distinct geographical pattern [6–8]. Co-occurrence of species has been postulated as a result of ecological differentiation, and not geographical isolation [9]. Based on the analysis of genome sequences, certain specialised metabolites occur in species-specific patterns for *S. tropica* and *S. arenicola* which could represent ecotype defining traits [10, 11]. However, these patterns have not been observed in *S. pacifica* [8]. At present, *Salinispora* strains are of particular interest because of their role as specialised metabolite producers [12–14]. Specialised metabolites includes many that possess important bioactivities and novel structures such as Lomaiviticin A, a potent antibiotic produced by *S. pacifica*, the anticancer agent Salinosporamide A produced by *S. tropica* and sioxanthin responsable for the characteristic orange pigmentation of colonies [15–17]. Although many efforts have been made to identify specialised metabolites and their associated biosynthetic gene clusters [18–20], there is little published regarding metabolism of *Salinispora* species [21, 22]. Growth capabilities help to define the phenotypic properties that define *Salinispora* species. Also, the identification of unique nutrient sources can aid the development of fermentation methods to increase the production of specific metabolites, and by optimizing fermentation conditions, the expression of dormant loci could be triggered.

Genome-scale metabolic reconstructions enable determination of functional capabilities of an organism [23, 24]. These metabolic models have been successfully used to predict growth rates and metabolic demands under different nutrients availability, thermosensitivity, for the interpretation of high-throughput data, guidance of strain design, and helping hypothesis-driven discovery [25–29].

Few manually curated actinomycete reconstructions are currently available despite their important role in drug discovery. Recently, the first manually curated genome-scale metabolic model for *Salinispora tropica* strain CNB-440^T^ was constructed [22]. The *i*CC908 model was based on physiological and biochemical information of primary and specialised metabolism pathways. The reconstruction enabled characterization of the metabolic capabilities for understanding and modeling the cellular physiology of this actinobacterium through integration and validation of experimental observations. The validation gave some insight about the missing context-specific information. Moreover, this model provides a starting point to produce Genome-scale models (GSMs) of closely related organisms such as other *Salinispora* species [30–32]. In continuation with efforts performed towards elucidating the metabolism of *Salinispora*, this study is focused on new insights into the metabolism of the three-identified species using constraints-based modeling. Here, we updated the model of *Salinispora tropica* strain CNB-440^T^ (from *i*CC908 to *i*CC926) where new genes and novel reactions have been added as more information about the genome and its annotation has become available [17]. GSMs were constructed for fully sequenced type strains of *S. arenicola*, and *S. pacifica*, strains CNH643^T^ and CNR-114^T^, respectively. These are the first manually curated genome-scale metabolic reconstructions for these *Salinispora* strains. We also constructed a *Salinispora* core model that contains the genes shared by 93 sequenced strains. Therefore, the GSMs cover the three-identified species and the core metabolic capabilities among *Salinispora* strains. Condition-specific models were used to predict and analyze metabolic capabilities of the three species under a minimal growth-supporting environment. Also, functional differences between the developed metabolic networks were identified to have a glimpse into the unique metabolic differences attributable to each *Salinispora* species.

## Methods

### Strains and media compositions

Six strains of *Salinispora* were tested for their ability to grow under a minimal growth-supporting environment as part of this study. *S. tropica* strain CNB-440^T^ was kindly provided by Dr. Paul Jensen (University of California San Diego, US). *S. arenicola* strain CNH-643^T^, and *S. pacifica* strains CNH-732, CNS-143, CNY-231, and CNS-055 were kindly provided by Dr. Michael Goodfellow (Newcastle University, UK). The strains were maintained on medium M1 [7] at 28.5 °C and 250 RPM and as glycerol suspensions (20%, *v*/v) at − 80 °C. M1 composition per liter of salt formulation: 10 g of starch (Sigma), 2 g of peptone (Sigma), and 4 g of yeast extract (Sigma). Medium M1 is also referred to as A1 [33, 34]. In this case, salt formulation SF2 was used for the media. This salt formulation has been previously described by Tsueng and Lam (2010).

A defined minimum medium (DMM) based on salt formulation SF2 [3] and marine broth composition was used to define minimum growth conditions. DMM contains 20 mM of a carbon source supplemented with a salt formulation. *Salinispora tropica* CNB-440^T^ was used as positive control to determine growth in DMM. DMM was modified to remove the sulfur source to determine if it supported growth, and if sulfur was accumulated as observed previously for *S. tropica* CNB-440^T^. Negative controls were done to discard toxicity problems. All experiments were done in duplicate. DMM formulation has been previously described in detail by Contador et al. (2015).

### Growth measurements

A protein extraction protocol was selected to determine the growth of the cultures over time [34]. Ultra-violet (UV) absorptions at 230 nm and 260 nm were measured using an Ultrospec 3000 UV/Visible spectrophotometer (Pharmacia Biotech). Total protein concentrations were calculated as follows: Total protein (ug/ml) = (183 x A_230_) – (75.8 x A_260_).

### Strain-specific metabolic network reconstructions

To create metabolic reconstructions to represent each *Salinispora* species, the gene sequence from the metabolic model for *Salinispora tropica* CNB-440^T^ was used to identify orthologs. The InParanoid algorithm version 8.0 [35] was selected as the orthology inference method. This BLAST-based method used a pairwise similarity score between two complete proteomes for constructing orthology groups, followed by a clustering step where a cluster is seeded by a reciprocally best-matching orthologous pair. InParanoid detects reciprocal best hits as well as in-paralogs (paralogs that arise from duplication after speciation). A score-cutoff of 40 bits, a sequence overlap of 0.5, a group merging cutoff 0.5 and in-paralog confidence cutoff of 0.05 was used as InParanoid settings. BLAST was downloaded from [36]. We studied 89 genomes of *Salinispora* strains with high quality draft genomes together with the type strains were selected to build an ortholog table. The ortholog table, and a heatmap of InParanoid scores are available in Additional file 1: Tables S1 and S2, respectively. (Additional file 1: Table S3) contains genome and available isolation metadata of each strain.

Reactions from *i*CC926 were added to the new models according to the ortholog table or if no genes were associated to the reaction. Reactions associated to missing orthologs were not considered. Gene-protein-reaction (GPR) associations were updated for each reaction based on the ortholog table. GPR associations provide information about which gene encodes which proteins and links the functional proteins to one or more enzymatic reaction. Additional reaction, and gene content was added from KBase [37], and KEGG [38]. For that purpose, ortholog predictions of *S. arenicola* CNH-643^T^ and *S. pacifica* CNR-114^T^ against *S. arenicola* CNS-205 were identified comparing two proteome from KBase’s methods [39]. A minimum sub-optimal best bidirectional hit (BBH) ratio of 90% was used. The KEGG database only has information related to *S. arenicola* CNS-205, and *Salinispora tropica* CNB-440^T^ strains. In addition, reactions from the KBase automatic model generator were evaluated and added to the reconstructions. All draft reconstructions were manually inspected, curated and validated to include essential biomass components, specialised metabolic pathways, and to permit computation of steady-state properties at a broad range of growth conditions such as minimal media. A well-established reconstruction protocol and curation tutorial was followed through the process [40]. Literature, and organism-specific and unspecific databases (e.g. KEGG, PATRIC, ExPASy and NCBI) were used to obtain information for gene annotation, pathway utilization and other physiological and phenotypical properties with particular focus on specialised metabolism. NCBI Locus Tags were used as gene IDs. The updated version of *i*CC908 was used to standardize metabolites and reaction identifiers and annotations. A list of the reactions and metabolites included in each reconstruction with corresponding references, associated genes, E.C. numbers and notes is available in Additional file 2. All genome sequences were downloaded from GenBank [41] on March 20, 2016. Mass and charge balances were verified using protocol described by Chan et al. (2017).

### Phylogenetic analysis

Phylogenetic estimation was carried out using the amino acid sequences of the proteins encoded by the core genes. Protein sequences were aligned at the amino acid level using MUSCLE [42]. Then, the alignments were concatenated using Geneious v11.1.2 [43]. Phylogenetic analyses were estimated using MEGA 7 [44] following multiple alignment performed with MUSCLE. Evolutionary distances were calculated and clustering determined under the maximum-likelihood method [45] using a gamma model of rate of heterogeneity and estimation of the proportion of invariant sites. The resultant tree topology was evaluated by a bootstrap analysis based on 100 resamplings.

### Biomass reactions

Biomass equations are critical components to predict growth rates. The biomass reaction from *i*CC926 was updated based on the macromolecular composition of each species to reflect specific physiology [4, 5]. Essential components and their fractional contributions were adapted from Borodina et al. (2005) as previously described [22]. The biomass equation of the Core model was developed based on precursor metabolites since most of the subunits of the macromolecules are not present [46]. The biomass equations are described in detail in the Additional file 3. Chemical formulae of biomass components were standardized to ensure that the biomass produced by the models have a molecular weight (MW) of ~ 1 g mmol^− 1^ [47].

### In silico growth conditions

Growth rates and metabolite production were predicted using Flux balance analysis (FBA) [48] from COBRA Toolbox [49]. Predictions were matched qualitatively to the experimental results reported in the literature and the experimental data generated in this work. In silico simulations were performed to predict the strain’s capacity to grow under minimal conditions. Details of DMM composition have been given previously [22]. DMM composition was simulated by allowing unlimited uptake of Ca^2+^, Cl^−^, Co^2+^, Cu^2+^, Fe^2+^, H^+^, H_2_O, Mg^2+^, Mn^2+^, K^+^, Na^+^, Mo^+ 7^ and Zn^2+^. The lower bounds of the respective exchange reactions were set to − 100 mmol gDw^− 1^ h^− 1^. The default carbon, nitrogen, phosphorous and sulfur sources were glucose, ammonium, phosphate, and sulphate. Default lower bounds were set to − 20 mmol gDw^− 1^ h^− 1^. To identify growth-supporting nutrients, the default carbon, nitrogen, phosphorous, and sulfur sources were removed one at a time by setting their lower bound to 0 mmol gDw^− 1^ h^− 1^. FBA was used to determine growth rates for each condition one at the time. Aerobic conditions were simulated by limiting the oxygen uptake rate to 20 mmol gDw^− 1^ h^− 1^ [50]. Viability threshold was set to 10% of the maximal growth rate under default conditions for computational identification of nutrients that support growth [51].

For production of sioxanthin by *Salinispora* strains, salinosporamide and sporolide by *S. tropica*, and lomaitivicin A by *S. pacifica*, growth rate was maximized and then used as an additional constraint to maximize the production of the specialised metabolites since they are produced alongside biomass. For simulation of in silico single gene knockouts, constraints were added by setting the bounds of the corresponding reaction(s), as defined by the GPR association(s), to 0 mmol gDw^− 1^ h^− 1^. The function *analyzeSingleGeneDeletion* [52] implemented in the COBRA Toolbox was used to compute the growth rate ratio between deletion strain and wild type to identify the set of essential genes for each model. A gene was classified as essential if the growth rate ratio was less than a cutoff (0.05). The flux of non-growth associated ATP maintenance (ATPm) was fixed at 3 mmol gDw^− 1^ h^− 1^ [22]. This reaction simulates the consumption of ATP by non-growth associated processes such as maintenance of electrochemical gradients. Simulation conditions and constraints used in the validation process have been previously described [22]. Uptake constraints for non-defined media were set to match the experimental conditions as closely as possible. All simulations were performed using the Matlab-based COBRA Toolbox. Gurobi™ Optimizer was employed as a linear programming solver.

### Structural analysis

To visualize relationships between the GSMs, binary matrices were generated to indicate: (a) whether a metabolic reaction is present or absent in a particular model, and (b) whether a gene is essential or not under a given growth environment. UpSet R package [53] was used to visualize exclusive reaction intersections between GSMs. Additionally, heat maps were generated using the ComplexHeatmap R package [54] with values aligned based on the calculated dendograms. Dendograms were generated based on euclidean distance metric, and completed-linkage clustering.

### Sampling

The Metabotools Toolbox protocol [26] was used to analyze context specific models. A sampling analysis was performed for the *Salinispora* models under DMM growth conditions and models were only able to produce sioxanthin as specialized metabolite since minimal conditions were evaluated. Models without blocked reactions were used as inputs. A threshold was set to 10e-6 for defining the active reaction set of each model. Reactions with fluxes less than this threshold were removed. The ACHR sampler implemented in the COBRA Toolbox [55] was used, gathering 5000 points in each file (nFiles = 100). In the initial phase, 3100 warmup points were generated, and 500 steps were skipped between two collected points.

## Results

### *i*CC926 and *Salinispora* models

**Fig. 1:**
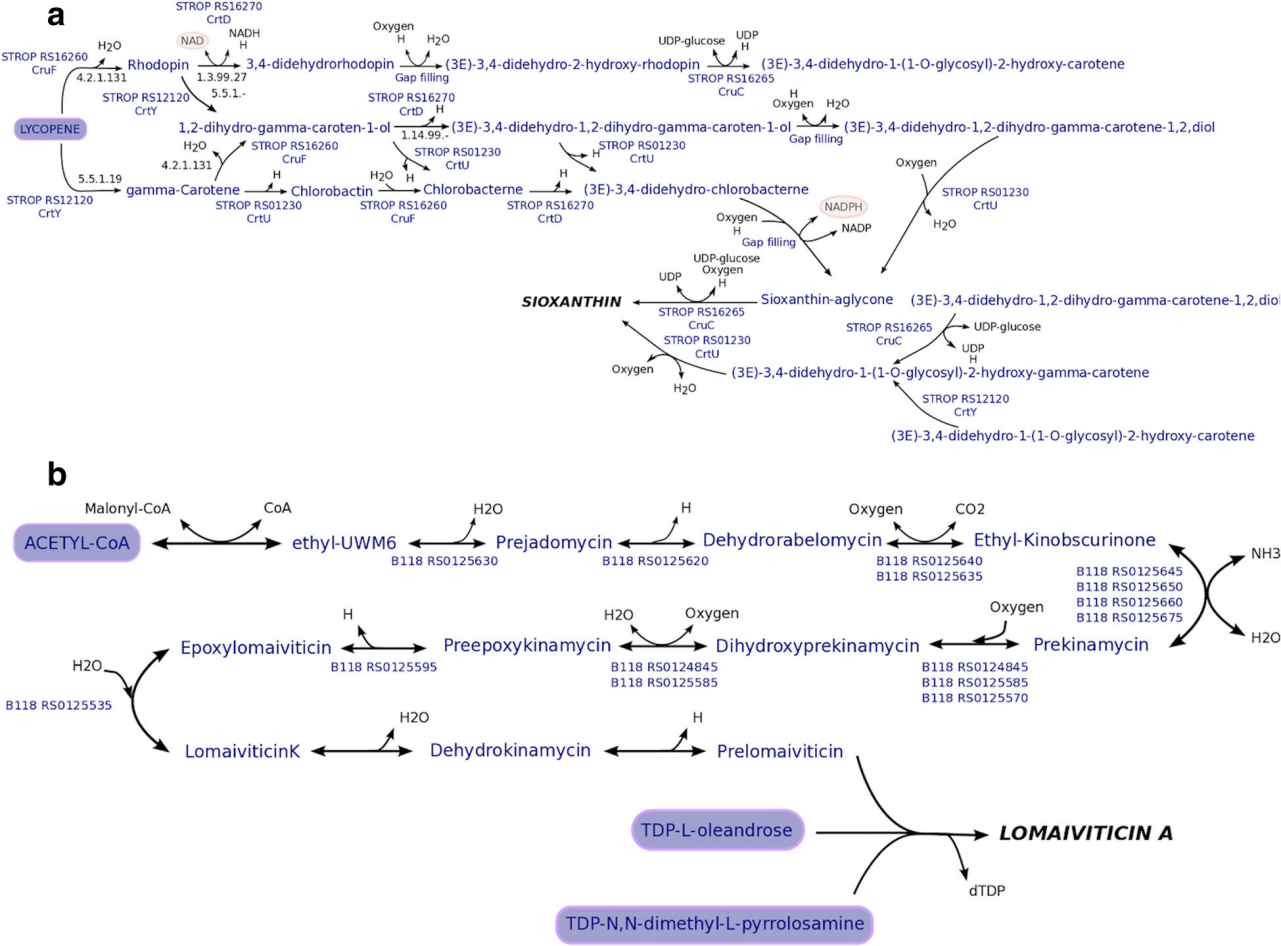
Overview of biosynthetic pathways of: (**a**) sioxanthin, and (**b**) lomaiviticin A

The content of the genome-scale metabolic model for *Salinispora tropica* strain CNB- 440^T^, *i*CC908 [22], was expanded and refined to use the new model as a starting point for the reconstruction of unique GSMs for the *Salinispora* strains and Core model. *i*CC926 is larger in scope than *i*CC908, containing 18 additional open reading frames (ORFs), and 52 additional reactions. The additional reactions are up to date with the content in KEGG [38], and literature. These reactions are involved in carbohydrate metabolism, amino acid metabolism, metabolism of cofactors and vitamins, lipid metabolism, specialised metabolism, and transport. The specialised metabolism was expanded since the carotenoid responsible for the orange pigmentation associated to *Salinispora* cultures has been identified and named Sioxanthin. Sioxanthin is a glycosylated compound derived from lycopene, and a biosynthetic pathway has been proposed [17]. However, only some enzymatic reactions in the biosynthesis of sioxanthin have been characterized [56]. Thus, BLAST and databases such as KEGG and BRENDA were used to find the most probable cofactors and metabolite participants of the uncharacterized reactions. Twenty reactions were identified to represent the biosynthesis of sioxanthin. The pathway was manually entered into the model. Figure 1a summarizes the information collected for the biosynthetic pathway of this specialised metabolite. Furthermore, 25 reactions were removed from *i*CC908 because they were found to be duplicates, and 146 reactions were modified. These modifications were related to EC number, subsystem, and/or GPR associations. In *i*CC926, twenty-one non-GPR reactions are present in the model related to amino acid metabolism since no auxotrophies for essential amino acids have been identified [22]. To assess the performance of *i*CC926, we compared model predictions to a recent study where the ability of *S. tropica* to use sole growth supporting sources on a defined minimum medium was evaluated [22] and by contrasting predictions in terms of growth/no growth to published results [3–5, 33, 57]. Ninety-seven different conditions were interrogated with an accuracy of 92.8% compared to 92.7% of *i*CC908. Growth conditions and constraints have been previously described in detail [22].

The *Salinispora* models developed in this study were constructed manually using *i*CC926 as a template. Type strains were selected to build GSMs since they define the main characteristics of each species. To define the minimum growth requirements, the strains were examined for their capacity to grow in a minimal growth-supporting environment. No strain-specific auxotrophies were identified experimentally on DMM. As observed previously [22], growth was detected in DMM without a sulfur source. Hence strains may be able to store sulfur. However, no additional experiments were done to study sulfur accumulation since it is not in the scope of the present study. Growth capability studies on different sulfur sources must take into consideration the probable storage of sulfur by the strains. No growth was detected in the negative controls. Growth curves are available in (Additional file 4: Figure S4a, and S4b). *Salinispora pacifica* CNR-114^T^ is able to grow on M1 (data not shown). However, the strain was not tested in DMM, and *S. pacifica* strain results were used to support growth in DMM. Pathways for amino acid biosynthesis and metabolism were gap filled in each specific case according to the experimental data available. Physiological and biochemical information of primary and specialised metabolism pathways was added to the *Salinispora* models. For *Salinispora arenicola* strain CNH-643^T^ model, *i*SACNH643, reactions were added to allow growth on L-proline, L-threonine, L-tyrosine, and D-salicin as sole carbon sources under aerobic conditions [4]. D-salicin was not a metabolite present in the draft model and was added together with the associated reactions to the reconstruction. However, the pathway is not well documented. Also, it was verified that galactose was not utilized as a carbon source. The galactose degradation pathway was not present in the draft model. L-alanine has been identified as sole nitrogen source [5], and growth is predicted by the model. L-glutamic acid was predicted as a nitrogen source, but experimental evidence shows the opposite [5]. Additional experiments are required to study possible missing regulatory constraints. In total, thirty reactions (not including transport or exchange reactions) were added without a gene association for amino acid biosynthesis and metabolism. On the other hand, despite transporters being poorly characterized as in *S. tropica*, different transport systems were added to allow interchange with the environment. The sioxanthin biosynthesis pathway was gap filled since orange pigmentation has been observed, and production was predicted on M1 medium. The biomass reaction was updated to reflect the physiology of the organism. However, details about biomass composition are scarse. Macromolecular content was calculated assuming a growth rate of 0.109 h^− 1^ [58] as in *i*CC926.

*Salinispora pacifica* strain CNR-114^T^ model, *i*SPCNR114, predictes growth on ISP5, and ISP7 media since transport reactions were added for asparagine and L-tyrosine [5]. L-glutamic acid and L-alanine were predicted as sole nitrogen sources. However, only L-glutamic acid has been reported to allow growth [5]. For *i*SPCNR114, seven reactions were added without a GPR association related to amino acid pathways excluding transport and exchange reactions. Macromolecular content was calculated assuming a growth rate of 0.048 h^− 1^ [58] since lower growth rates were observed for the *S. pacifica* strains compared to the other two *Salinispora* species in the complex medium M1. As stated above, the biomass reaction was updated to reflect the physiology of this strain. In this case, the phospholipid biosynthesis equation was modified based on the chemotaxonomic analysis done by Ahmed et al. [5]. Phophatidylmethylethanolamine, and phosphatidylinositol dimannosides were added to the reaction. The Sioxanthin pathway was added to the reconstruction since orange pigmentation was observed in complex medium M1. Three reactions without GPR associations were added for sioxanthin production. *i*SPCNR114 contains an additional specialised metabolite, Lomaiviticin A. Lomaiviticin A is a member of the angucycline family of aromatic polyketides, and exhibit a potent antiproliferative antimicrobial activity [16]. Biosynthetic pathways have been proposed for this compound with different levels of detail [59, 60]. As stated above, the information on the uncharacterized steps was added to the model based on bioinformatic tools. Figure 1b summarizes the pathway. For Lomaiviticin A production, 27 reactions and 24 metabolites were added to the model.

Overall, 23 and 19 different conditions including DMM experiments and published data were tested during the validation process for the models of *S. arenicola* and *S. pacifica* with accuracies of 96 and 95%, respectively. The predicted growth rates and specialised metabolite production for each condition can be found in Additional file 5. The validation process allow capturing strain-specific metabolic capabilities.

### Characteristics of the *Salinispora* core model

The Core *Salinispora* metabolic model is a subset of the genome-scale metabolic reconstruction *i*CC926. The subset was selected according to the genes that were conserved across 93 sequenced *Salinispora* strains defining the core metabolic capabilities among all of the strains. The genome sequences were derived from 10 *S. tropica*, 45 *S. arenicola*, and 38 *S. pacifica* strains. Isolation and genome metadata are available in (Additional file 1: Table S3). Non-GPR reactions were added to the core model only if they were predicted to be essential for aerobic growth on complex medium to reduce the number of reactions without GPR associations. Additionally, the non-clustered gene arrangement responsible for carotenoid biosynthesis in *Salinispora* has been predicted to be present in all of the *Salinispora* strains whose genome is currently available [17, 56]. To the best of our knowledge a case to demonstrate the contrary has not been reported. Therefore, the sioxanthin pathway was added to the model. However, sioxanthin is not produced since a precursor of the carotenoid pathway, geranylgeranyl diphosphate, is not produced by the model. No additional reactions were added to solve the sioxanthin production. The core biomass reaction does not include phophatidylmethylethanolamine, and phosphatidylinositol dimannosides since these metabolites have only been reported in the macromolecular composition of *S. pacifica* [5], and no additional evidence is available to support their presence in all the strains.

**Fig. 2:**
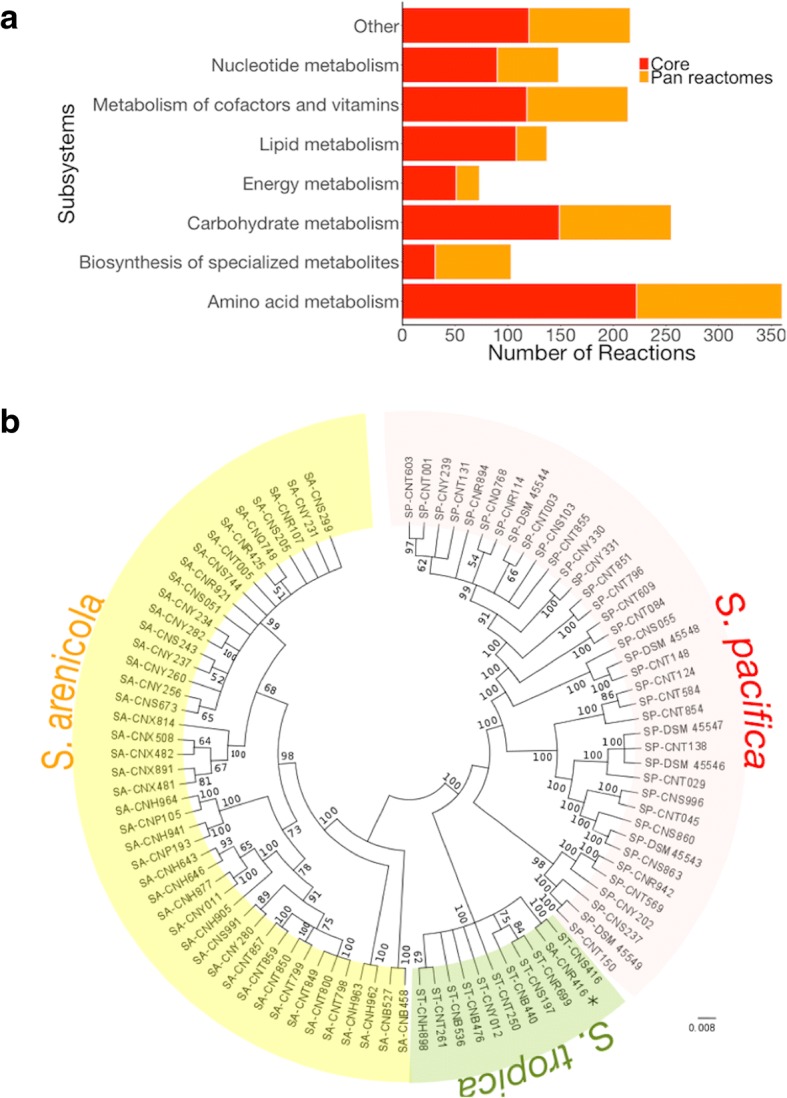
Core content of *Salinispora* species (**a**) Distribution of functional subsystems in Core and Pan reactomes. **b** Phylogenetic tree based on alignment of the concatenated amino acid sequences of the proteins encoded by the core genes. *S. tropica*, *S. arenicola*, and *S. pacifica* clades are highlighted in green, yellow, and pink respectively. The horizontal bar at the base of the figure represents 0.008 substitutions per site. The percentages that support the branches of the tree are indicated based on 100 re-sampled datasets (only values above 50% are given)

The content of the core model was compared to the pan metabolic capabilities among the three *Salinispora* models developed. The pan metabolic capabilities are defined by the reaction and gene sets present in all the *Salinispora* models. The reactions and genes present in the Core model represent on average 68 and 70% of the content of reactions and genes in the three models, respectively. The remaining reactions and genes are part of the unique metabolic capabilities of each strain. These results are comparable to previous studies of multiple GSMs [31, 32]. Table 1 summarizes key properties of the Core and *Salinispora* models. The distribution of reactions in the core and pan reactomes by functional subsystems is shown in Fig. 2a. A Fisher’s exact test was used to establish that differences observed in the conservation across the functional subsystems are statistically significant with *p*-value < 0.05 (8.9E-3). The most conserved subsystems were lipid metabolism (79%), energy metabolism (70%), amino acid metabolism (62%), and nucleotide metabolism (61%). These reactions are associated to oxidative phosphorylation, and essential components for cell growth as was defined by the biomass reactions and the revised literature. As was expected, the biosynthesis of specialised metabolites was the less conserved subsystem in the core reactome (30%). Specialised metabolites have been mentioned as unique traits that help to differentiate each species [2]. Additionally, core genes were evaluated as phylogenetic markers and a concatenated phylogeny was generated. Figure 2b shows congruent species phylogenies with three supported clades where *S. pacifica* clade is a sister group to *S. tropica*. These clades are consistent with information from literature [8, 61]. The only exception was *S. arenicola* CNR-416 which is placed in the *S. tropica* clade.

**Table 1:** *Salinispora* strain-specific metabolic model statistics comparison

	*i*CC926	*i*SACNH643	*i*SPCNR114	*i*Core
Total reactions	1530	1112	1363	876
Metabolic conversions	1188	941	1171	773
Reactions with ORF assignments	1039	826	1112	745
% of reactions with ORF	87	88	95	96
Reactions without ORF	149	115	59	28
% of reactions without ORF	13	12	5	4
Transport reactions	207	103	124	66
Transport reactions with ORF assignments	60	56	58	49
% of transport reactions with ORF	29	54	47	74
Exchange reactions	135	68	68	37
Number of ORFs	926	647	928	568
Number of metabolites	1321	1089	1292	920

### Structural analysis of the reconstructions

**Fig. 3:**
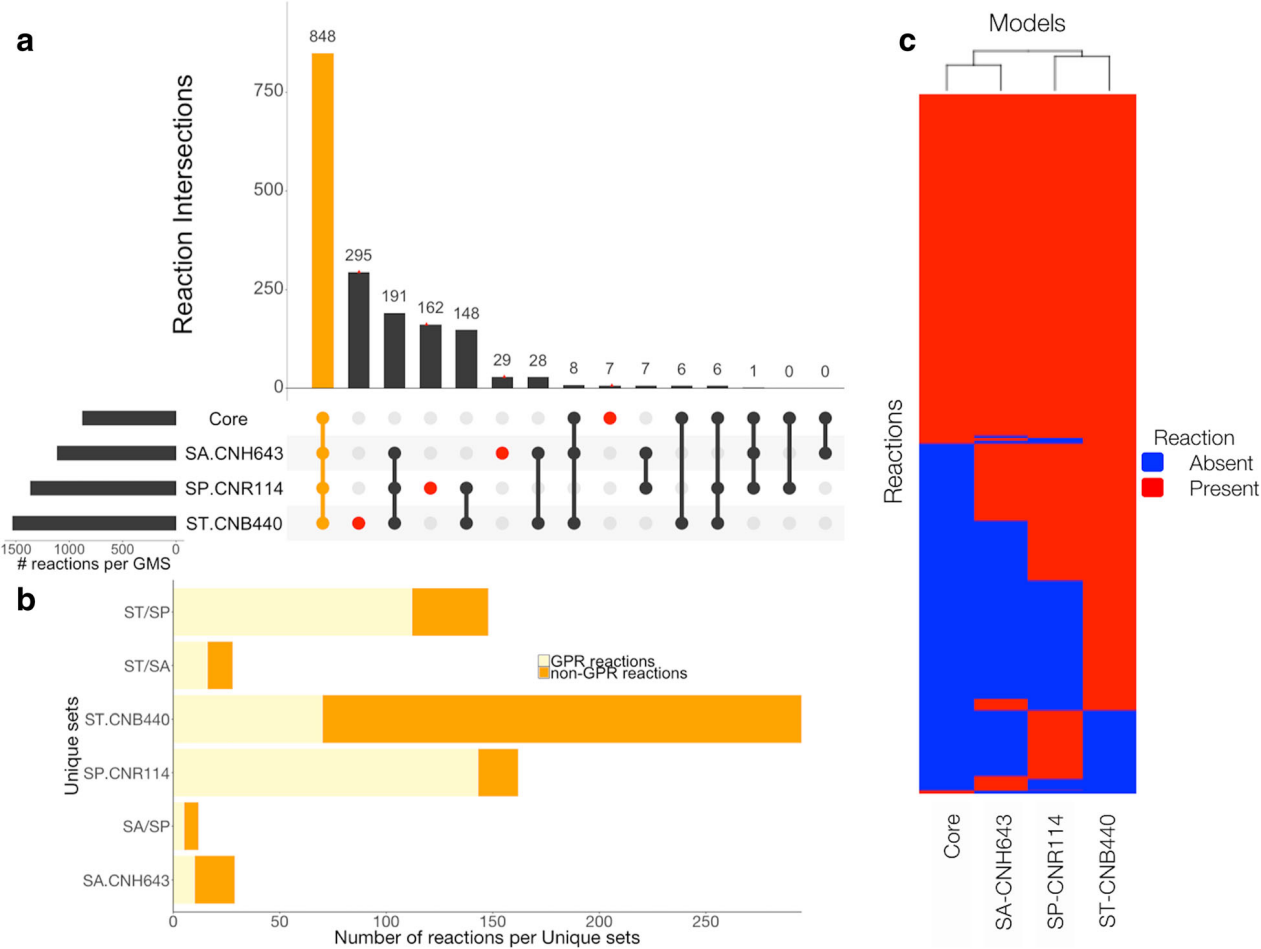
Reaction content across *Salinispora* models (**a**) Upset plot indicating shared and distinct reaction content between models. The unique sets of reactions for each GSM are highlighted by red circles. Intersections between the four models are indicated in orange. **b** Number of reactions with and without GPR associations in each distinct set of reactions. **c** Clustering of species by metabolic reactions. Rows represent individual reactions, and columns represent models. Heat map describes differences in reaction content across *Salinispora* models. Reactions absent in individual models are indicated in blue

As previously observed, unique sets of reactions and genes are responsible for the extra metabolic capabilities specific to each strain. To visualize the shared and distinct content between models, an Upset plot was generated [53]. As in Venn diagrams, Fig. 3a shows the exclusive intersections, and shared reactions between the models. For simplicity, *i*CC926, *i*SACNH643, and *i*SPCNR114 will be referred to hereafter as ST-CNB440, SA-CNH643, and SP-CNR114, respectively. From the graph, 848 reactions are shared between the four models. These reactions include both GPR and non-GPR associated reactions. Furthermore, it is observed that 191 reactions are absent in the Core model, and shared by the rest of the models. The 7 unique reactions of the Core model are related to the biomass reaction. On the other hand, ST-CNB440 presents the higher number of unique reactions among the four models while SA-CNH643 is the less distinct strain.

Since not only the magnitude of the sets is interesting, unique sets of reactions and intersections were analyzed to explore differences between reconstructions. The first step was to verify that these distinctive sets were not a major consequence of the gap filling associated to the biomass reactions, and the validation process. Reactions were classified according their GPR associations (GPR and non-GPR reactions). GPR associations determine the set of metabolic reactions encoded in the genome. From Fig. 3b, it is observed that the distinctive reactions associated to each GSM and shared sets among models are mainly due to genome sequence data with an average of 77% of reactions with a GPR association except for the ST-CNB440 and SA-CNH643 reconstructions with only 24 and 35% of their reactions with GPR associations, respectively. In the first case, additional reactions were added due to experimental evidence about the ability of the strain to use sole growth supporting sources [22]. Then, most of these reactions are a result of the gap-filling process performed to ensure that the reconstruction was able to reproduce the in vivo phenotypes. An extensive phenotypic profiling of the other two species of *Salinispora* was not available at the time of the analysis. In the case of SA-CNH643, 32% of the non-GPR associated reactions are related to biomass and transport. The unique set of SP-CNR114 shows the highest number of GPR associations with 88%. Moreover, the distribution of the unique, and shared reactions by the functional subsystem was found not to be similar. The majority of these reactions are involved in transport (48%), amino acid metabolism (30%), and carbohydrate metabolism for ST-CNB440, SA-CNH643, and SP-CNR114, respectively. Amino acid and carbohydrate metabolism accounted for 31 and 21% of the shared reactions between ST-CNB440, and SA-CNH643. Twenty five percent of the shared reactions by ST-CNB440, and SP-CNR114 are involved in the metabolism of vitamins and cofactors. Meanwhile, most of the reactions shared between SA-CNH643, and SP-CNR114 are related to lipid metabolism (29%). These shared sets defined common features between species. The distribution of sets by subsystems can be found in Additional file 5: Figure S5. Clustering of species by model reaction content revealed that SA-CNH643 and the Core are the most similar followed by SP-CNR114 and ST-CNB440 as indicated by Fig. 3c. This result suggests that SP-CNR114 and ST-CNB440 have a similar metabolic content.

### Condition-specific models for DMM

**Fig. 4:**
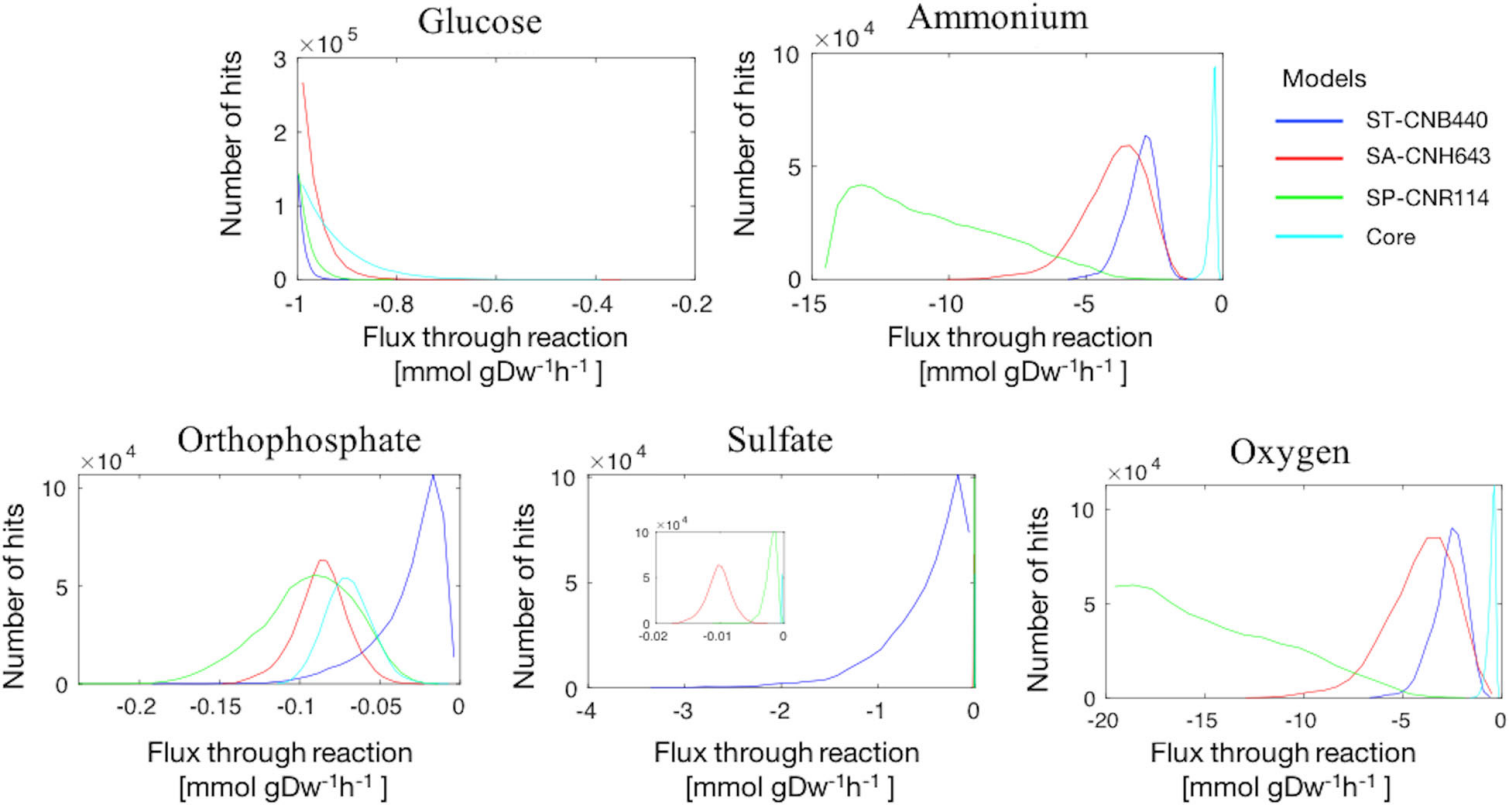
Flux distribution histograms for essential nutrient uptake reactions in the *Salinispora* models. Distributions for ST-CNB440, SA-CNH643, SP-CNR114 and Core are in blue, red, green, and cyan respectively. The x axis of the histograms indicates the magnitude of the fluxes. The y axis of the histograms indicates the probability density. Bidirectional reactions with flux in the reverse direction are represented by negative values in the histograms. Sulfate plot shows a magnification of the distributions for SA-CNH643, SP-CNR114 and Core

Next, condition-specific models were used to determine if the identified differences in reaction content between models gave rise to different functional predictions of essential nutrient usage by each species. For this purpose, the genome-scale metabolic reconstructions for *Salinispora* strains and core model were converted into computational models to allow the simulation of growth in minimum medium. Condition-specific models were derived from the active reactions of these computational models in DMM. Reactions unable to carry flux were identified through flux variability analysis (FVA) [62], and removed from the models. DMM composition was used to restrict the metabolite uptakes. Exchange profiles of the models showed oxygen, and essential nutrient requirements as have been observed experimentally in previous work [22] and this study (Additional file 4). Essential nutrients cover carbon, nitrogen, sulfur, and phosphorous sources as defined by DMM composition. Additionally, secretion patterns were partially overlapping. It has been mentioned that differences in the exchange profiles could suggest distinct metabolic states [63]. To interrogate the functional differences predicted by the four contextualized models, a sampling analysis of the solution space of each model was performed. Even when the metabolomic data was scarce, sampling revealed different nutrient utilization under DMM growth conditions as shown in Fig. 4. Only reactions with experimental evidence were illustrated. The histograms represent the probability that certain reactions can have a particular flux rate. Table 2 summarizes the median values of the sampling results. A comparison of the distributions and medians revealed a shift indicating a difference in the use of essential nutrients by the four contextualized models. A slight shift in the distribution of glucose was observed, suggesting a similar utilization of glycolysis by the models. SP-CNR114 has higher median flux through oxygen uptake reaction which could be related to a higher oxidative metabolism. However, additional experiments are required to support this hypothesis. As stated above, differences in growth rates were experimentally observed between the strains. Models could explain in more detail these differences if more data becomes available to add additional constraints such as secretion profiles.

**Table 2:** Median flux values of essential nutrient uptake reactions

Uptake reaction	ST-CNB440 median [mmol gDw^−1^ h^− 1^]	SA-CNH643 median [mmol gDw^−1^ h^−1^]	SP-CNR114 median [mmol gDw^− 1^ h^− 1^]	Core median [mmol gDw^− 1^ h^− 1^]
Glucose	−0.99191	−0.97725	− 0.98539	− 0.94792
Ammonium	−2.929	−3.8176	−10.491	− 0.33548
Orthophosphate	−0.02465	− 0.0850	− 0.0928	−0.06996
Sulfate	−0.3839	−0.01013	− 0.001786	−0.00036
Oxygen	−2.5176	−3.8943	− 15.145	−0.4596

### Prediction of essential genes

**Fig. 5:**
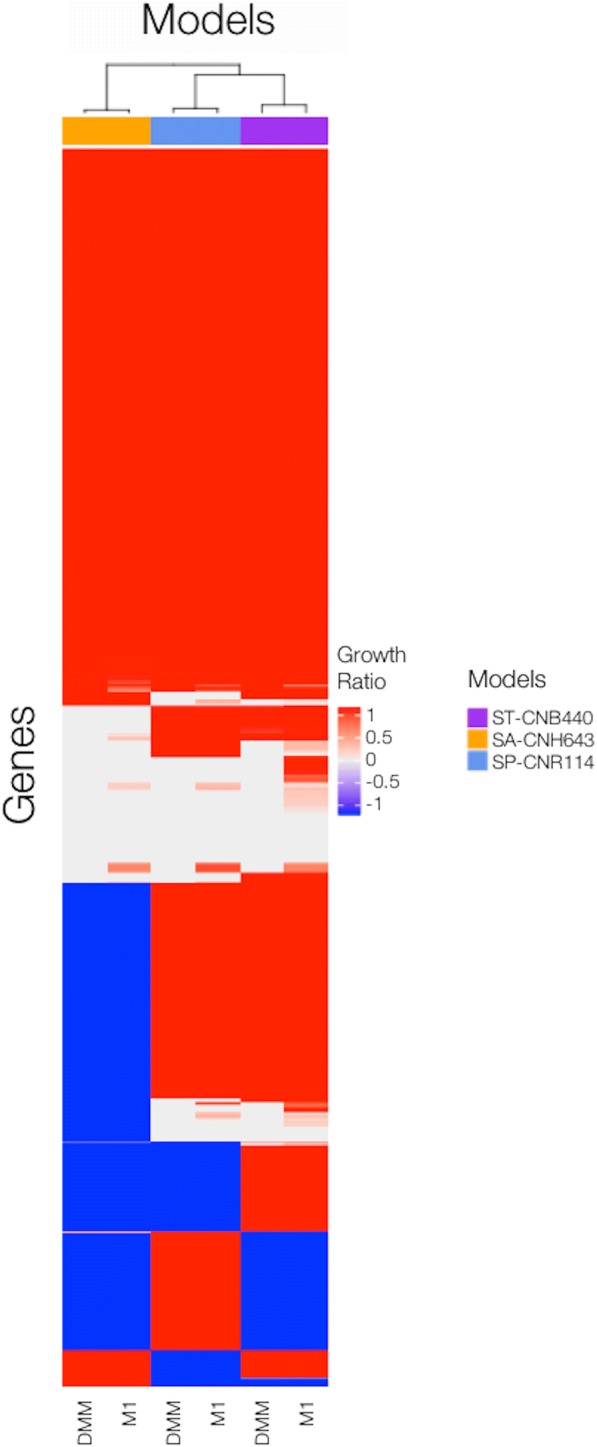
Clustering of species by effect of single gene deletion across models. Growth ratio is defined by maximal growth rate ratio between deletion strain and wild type strain. Genes absent in individual models have assigned a growth ratio value of − 1. Essential genes were defined by growth ratio less than 0.05. Strains are clustered based on effect of the deletion in a given growth environment. Rows represent individual genes, and columns represent DMM, and M1 predictions for each model

Finally, gene content was evaluated to identify genetic differences between species. Models were used to identify essential genes in DMM and complex medium M1. A gene was classified as essential if one or more reactions associated to this gene need to carry a flux to satisfy a particular objective function, i.e., the gene is necessary for in silico growth in the given conditions. Results of simulations were categorized according to the effect of the deletion compared to the wild-type strain (Additional file 1). Figure 5 describes the outcome of single gene deletion in a given growth environment across *Salinispora* models. Genes that were absent in models were assigned a growth ratio value of − 1. The analysis yielded 96 and 42 shared lethal gene deletions for the models in DMM and M1, respectively. A unique set of essential genes was identified for ST-CNB440 (4 genes), SA-CNH643 (31 genes), and SP-CNR114 (10 genes) in DMM growth conditions. Three of the unique essential genes of the ST-CNB440 model were only present in this model. For complex medium, 3, 36 and 22 distinct sets of essential genes were identified for ST-CNB440, SA-CNH643, and SP-CNR114, respectively. Clustering of species by the effect of single gene deletions in the models revealed that the SA-CNH643 model is more different from the other models. ST-CNB440, and SP-CNR114 models are still more metabolically similar when considering predicted essential genes. Phylogenetic studies have shown that *S. pacifica* strains form a distinct phylogenetic lineage that is a sister to *S. tropica* [9]. Also, it was observed that deletions of certain genes were lethal only in specific models as in the case of B118_RS0101230. This gene is lethal in the SP-CNR114 model, but its ortholog is not, due to the presence of two isozymes for the same reaction in the ST-CNB440 model. Alternative pathways also play a role. Reactions associated with essential genes drive gene essentiality. These reactions were identified and most of them are involved in the synthesis of essential components such as amino acids, vitamins and cofactors like riboflavin, and Coenzyme A as well in the utilization of oxygen. However, genetic differences must be studied in detail using experimental data to discard errors in the assignment of the GPR associations. Essential genes and associated reactions can be found in Additional file 1**.**

## Discussion

In continuation with efforts regarding the study of the metabolism of *Salinispora* species, we focused on gaining new insights into the metabolic capabilities of the three-identified species of *Salinispora* using constraints-based modeling. Here, models were constructed for each of the three currently identified *Salinispora* species. The selection of type strains to build GSMs make them the ideal templates for future reconstructions of *Salinispora* strains or closely related organisms. These templates will allow the study of the metabolism of organisms with high biotechnology potential such as *Salinispora arenicola* strain CNS-205. *S. arenicola* CNS-205 has promising biotechnological potential since it produces the unusual antiinflamatory cyclic peptide cyclomarin A [64]. Also, a core model representing the conserved metabolic capabilities of *Salinispora* was built. From the analysis of the core model, it was found that specialised metabolites appear to be a distinct trait between *Salinispora* strains even when they share a common carotenoid. This is in agreement with the evidence described through molecular networking and genome mining studies [20]. It is also hypothesized that conserved pathways are mostly related to similarity in lipid composition and chemotaxonomy of *Salinispora* strains. In vitro studies were performed to explore additional functional differences between strains on defined minimum medium (DMM). Strain-specific auxotrophies were not identified. Additionally, the phylogeny generated using the core genes was consistent with the species phylogeny in the literature. However, one exception was observed with *S. arenicola* CNR-416 that was included in the *S. tropica* clade. Then, analysis and selection of phylogenetic markers are important to avoid incongruent associations.

GSMs were validated and analyzed to explore the differences between the reconstructions by growth phenotypes. Despite the structural similarities between the models, results suggest that models are able to describe different metabolic states of *Salinispora* strains. Functional differences between the developed metabolic networks were identified to have a glimpse into the cell metabolism that are different in each *Salinispora* species. Disagreements with experimental results indicate potential errors in the reconstructions or missing regulatory constraints. Additional knowledge is necessary to better represent metabolite consumption and byproduct release as in the case of the specialised metabolites. Transport systems are not well documented, and energy costs could be underestimated. Since knowledge about *Salinispora* species is continually evolving, new data can be incorporated into the reconstructions to better represent the metabolism of each species. The overall accuracies of ST-CNB440, SA-CNH643, and SP-CNR114 models were 92.8, 96 and 95%, respectively. Additional phenotypic profiling is required to test different nutrient requirements. Phenotypic microarrays are a good alternative to study the ability of strains to use different nutrient sources [65, 66]. Experimental evidence reported in this study, and in previous work [22] suggests that *Salinispora* strains may be able to store sulfur. The ability of the strains to use different sulfur sources must take into consideration these findings.

**Fig. 6:**
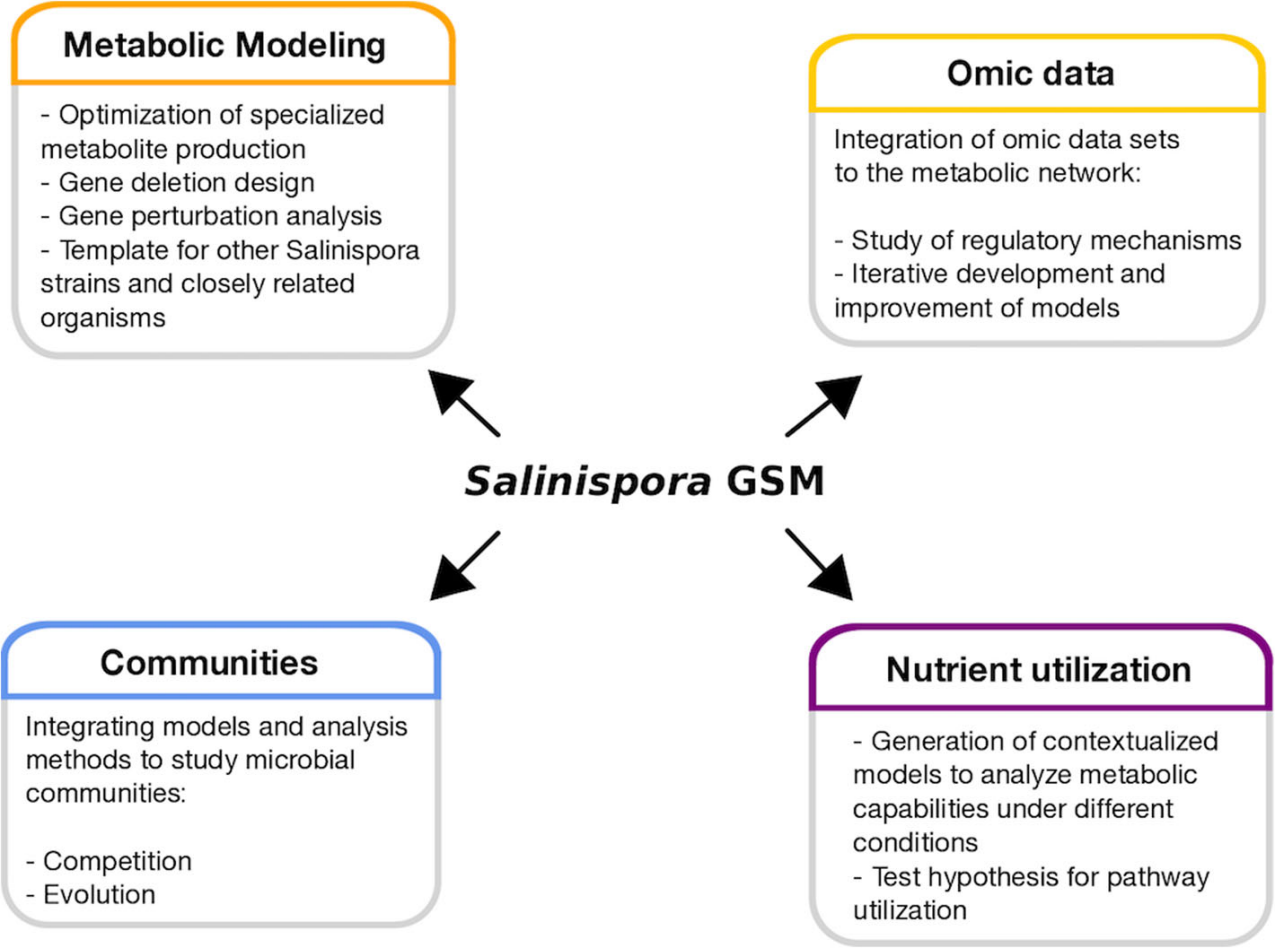
Applications of *Salinispora* models to study metabolic functionalities of the *Salinispora* genus

Condition-specific models were used to predict and analyze metabolic capabilities of the three-species using a qualitative approach. Models allow assessment of the metabolic characteristics of the cell when integrating environment conditions. The contextualized models predicted different usage of essential nutrients on DMM. This could explain the differences observed in growth rates on DMM (Additional file 4). Clustering of species by effect in single gene deletions and reaction content in models revealed that the ST-CNB440, and SP-CNR114 models are more metabolically similar. Figure 6 summarizes applications of *Salinispora* models after analyzing the metabolic capabilities of the networks. These applications cover from integration of omic data (e.g. transcriptomic, metabolomic, etc) to the study of microbial communities. Specialised metabolites are involved in the microbial interactions with the environment and other microorganisms. The study of *Salinispora* metabolism is still in the early stages, however the use of metabolic models could help to gain new knowledge of this marine actinomycete and the production of specialised metabolites.

## Conclusions

This study shows that strain-specific models of *Salinispora* can help to better understand the metabolism of *Salinispora* strains, and gain more knowledge about the physiology of the different species. *Salinispora* models would help researchers to establish links between genetic data and metabolic phenotypes. Additionally, the models developed can be used to systematically analyze the essential growth capabilities of *Salinispora* metabolism that delineate the adaptation process and aid researchers to guide and enhance the production of specialised metabolites.

## Additional files

Additional file 1: **Table S1.** contains ortholog data used to build the Core model (worksheet1). **Table S2.** contains a heat map of InParanoid scores (worksheet2). **Table S3.** contains genome and available isolation metadata of *Salinispora* strains in Core (worksheet3). References used to extract data related to isolation metadata are listed in the table. **Table S4.** contains list of conserved and not conserved reactions in each mode (worksheet4). **Tables S5**, **S6** and **S7.** contain reactions associated to essential genes in ST-CNB440, SA-CNH643 and SP-CNR114 models under DMM growth conditions, respectively (worksheet5). **Table S8, S9** and **S10.** contain reactions associated to essential genes in ST-CNB440, SA-CNH643 and SP-CNR114 models under CM growth conditions, respectively (worksheet6). **Table S11.** contains growth rate ratio between deletion strain and wild type strains for each model under DMM and CM growth conditions (worksheet7). (XLSX 2620 kb)

Additional file 2: Excel files with list of reactions, metabolites, and genes included in each reconstruction with corresponding references, associated GPR, E.C. numbers and notes. Mat files for each *Salinispora* model built in this work. (MAT). (ZIP 1303 kb)

Additional file 3: Details of biomass equations used for Core (worksheet1), *Salinispora arenicola* (worksheet2) and *Salinispora pacifica* (worksheet3) models. References are included in the file. (XLSX 67 kb)

Additional file 4: **Figure S4a.** Growth curves of *Salinispora* strains in DMM without sulfur sources. Sulfur source was not included in the medium to examine the ability of strains to store sulfur as was observed in the case of *S. tropica* CNB-440^T^ by Contador et al. 2015. Sulfur must be supplemented to the medium after the depletion of the accumulated sulfur. All experiments were done in duplicate. Error bars represent standard deviation. **Figure S4b.** Growth curves of *Salinispora* strains in DMM without carbon sources. (ZIP 214 kb)

Additional file 5: **Table S1 to S8.** Predicted growth rates and specialised metabolite production used in the validation process of the models. Uptake constraints for non-defined media were set to match the experimental conditions as closely as possible. References to published studies used in the validation are included in the file. **Figure S5.** Distribution of unique sets of reactions by subsystems. (a) ST-CNB440; (b) SA-CNH643; (c) SP-CNR114; (d) ST-CNB440/SA-CNH643; (e) ST-CNB440/SA-CNH643. Sets with 20 or more reactions were represented. (DOCX 492 kb)

## Abbreviations

DMM: Defined minimum medium
FBA: Flux balance analysis
FVA: Flux variability analysis
GPR: Gene-protein-reaction
GSM: Genome-scale models
