# Supplementary figures and images for: Use of genome-scale models to get new insights into the marine actinomycete genus Salinispora
Source: BMC Syst Biol. 2019 Jan 21;13:11. doi: 10.1186/s12918-019-0683-1 (PMC6341766; doi:10.1186/s12918-019-0683-1)

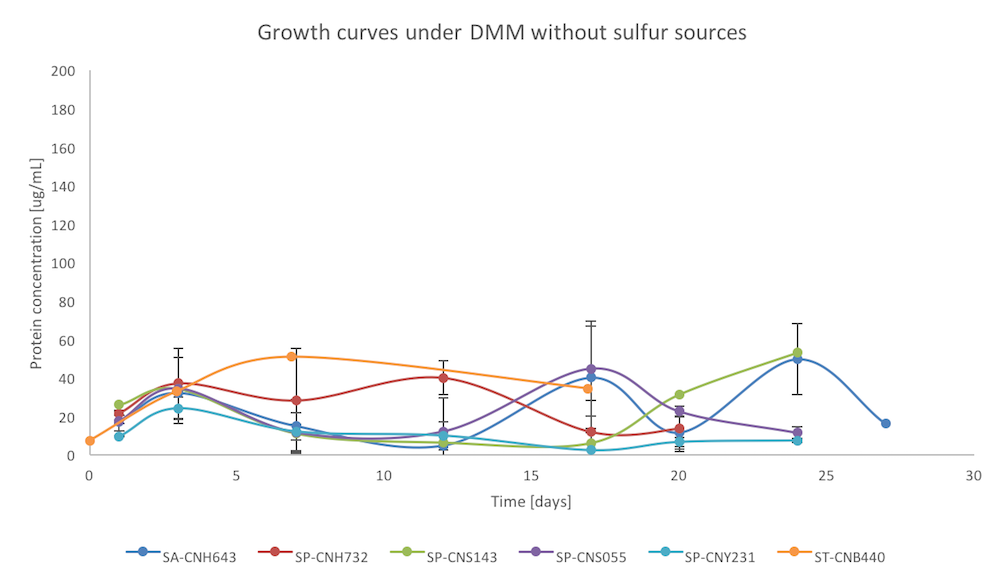

Supplement: Supplementary file 4 — Figure S4a. Growth curves of Salinispora strains in DMM without sulfur sources. Sulfur source was not included in the medium to examine the ability of strains to store sulfur as was observed in the case of S. tropica CNB-440T by Contador et al. 2015. Sulfur must be supplemented to the medium after the depletion of the accumulated sulfur. All experiments were done in duplicate. Error bars represent standard deviation. Figure S4b. Growth curves of Salinispora strains in DMM without carbon sources. (ZIP 214 kb) [file 12918_2019_683_MOESM4_ESM.zip › FigureS4a.png]

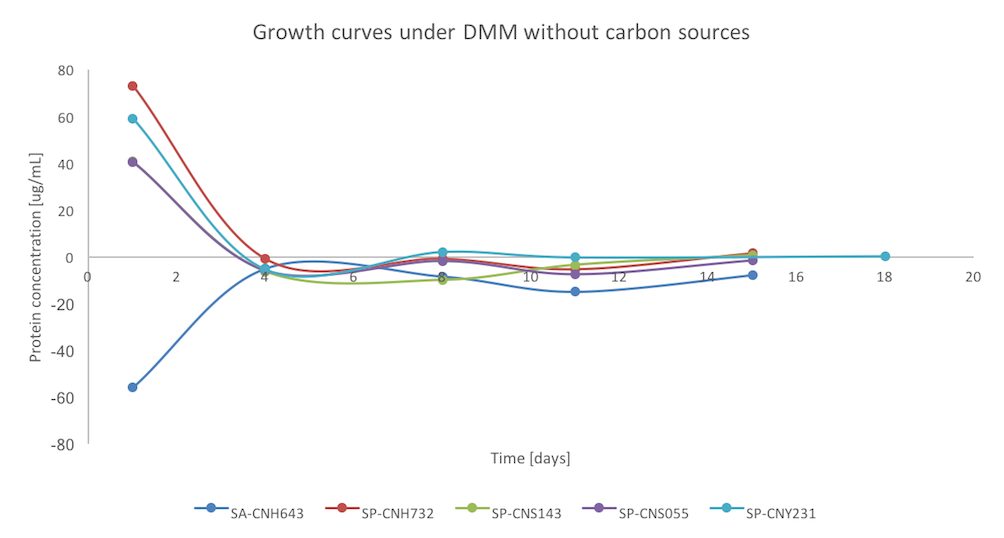

Supplement: Supplementary file 4 — Figure S4a. Growth curves of Salinispora strains in DMM without sulfur sources. Sulfur source was not included in the medium to examine the ability of strains to store sulfur as was observed in the case of S. tropica CNB-440T by Contador et al. 2015. Sulfur must be supplemented to the medium after the depletion of the accumulated sulfur. All experiments were done in duplicate. Error bars represent standard deviation. Figure S4b. Growth curves of Salinispora strains in DMM without carbon sources. (ZIP 214 kb) [file 12918_2019_683_MOESM4_ESM.zip › FigureS4b.png]
