# Supplementary material for: Use of genome-scale models to get new insights into the marine actinomycete genus Salinispora
Source: BMC Syst Biol. 2019 Jan 21;13:11. doi: 10.1186/s12918-019-0683-1 (PMC6341766; doi:10.1186/s12918-019-0683-1)
Supplement: Supplementary file 5 — Table S1 to S8. Predicted growth rates and specialised metabolite production used in the validation process of the models. Uptake constraints for non-defined media were set to match the experimental conditions as closely as possible. References to published studies used in the validation are included in the file. Figure S5. Distribution of unique sets of reactions by subsystems. (a) ST-CNB440; (b) SA-CNH643; (c) SP-CNR114; (d) ST-CNB440/SA-CNH643; (e) ST-CNB440/SA-CNH643. Sets with 20 or more reactions were represented. (DOCX 492 kb) [file 12918_2019_683_MOESM5_ESM.docx]

**Validation of Core and *Salinispora* models**

**Table S1:** Simulations of Core model under different growth conditions.

|  | **Medium** | **Growth rate** | **Growth rate** | **References** |
| --- | --- | --- | --- | --- |
|  |  | ***in silico*** | **in vivo** |  |
|  |  | **1/h** |  |  |
| **1** | Medium without Carbon source | 0.000 | no growth | Contador et al 2015 |
| **2** | Medium without Nitrogen source | 0.000 | no growth | Contador et al 2015 |
| **3** | Medium without Sulfur source | 0.000 | no growth | Contador et al 2015 |
| **4** | Medium without Phosphate source | 0.000 | no growth | Contador et al 2015 |
| **5** | Glucose-Minimal Media Anaerobic | 0.000 | no growth | Ahmed et al 2013, Maldonado et al 2005 |
| **6** | Glucose-Minimal Media Aerobic | growth | growth | Ahmed et al 2013, Maldonado et al 2005 |
| **7** | Starch-A1 medium | 0.1346 | growth | This work |
| **8** | ISP2 (glucose-malt-yeast extract agar) | growth |  | Ahmed et al 2013 |
| **9** | ISP5 (glycerol-asparagine agar) | growth |  | Ahmed et al 2013 |
| **10** | ISP4 (inorganic salts-starch agar) | growth |  | Ahmed et al 2013 |
| **11** | ISP3 (oatmeal agar) | growth |  | Ahmed et al 2013 |
| **12** | ISP6 (peptone-yeast extract-iron agar) | growth |  | Ahmed et al 2013 |
| **13** | ISP1 (tryptone yeast extract agar) | growth |  | Ahmed et al 2013 |
| **14** | ISP7 (tyrosine agar) | growth |  | Ahmed et al 2013 |

**Table S2:** Predicted growth rates by iCC926 under different growth conditions.

|  | **Medium** | **Growth rate** | **Growth rate** | **References** |
| --- | --- | --- | --- | --- |
|  |  | ***in silico*** | **in vivo** |  |
|  |  | **1/h** |  |  |
| **1** | Glucose-Minimal Media | 0.4118 |  | Alam et al 2011 |
| **2** | Glucose-Minimal Media | 0.1489 | growth | Maldonado et al 2005 |
| **3** | Glucose-CM-SS | 0.5714 | 6 (% PCV) | Tsueng et al 2008 |
| **4** | Glucose-CM-SF1 | 0.5533 | 6 (% PCV) | Tsueng et al 2008 |
| **5** | Glucose-CM-SF2 | 0.5533 | growth | Contador et al 2015 |
| **6** | Glucose-CM-not salts | 0.5533 | no growth | Maldonado et al 2005 |
| **7** | Starch-SS | 0.9342 | growth | Lechner et al 2011 |
| **8** | Starch-SF1 | 0.9160 | +++, 5.97 mg/ml | Tsueng and Lam 2008b; Tsueng and Lam 2010 |
| **9** | Starch-SF2 | 0.9160 | 7.15 mg/ml, 6.45mg/ml | Tsueng and Lam 2008b; Tsueng and Lam 2010 |
| **10** | ISP2 (glucose-malt-yeast extract agar) | 1.288 | +++ | Ahmed et al 2013 |
| **11** | ISP5 (glycerol-asparagine agar) | 0.8571 | +++ | Ahmed et al 2013 |
| **12** | ISP4 (inorganic salts-starch agar) | 0.4467 | ++ | Ahmed et al 2013 |
| **13** | ISP3 (oatmeal agar) | 0.8809 | +++ | Ahmed et al 2013 |
| **14** | ISP6 (peptone-yeast extract-iron agar) | 0.0044 | no growth | Ahmed et al 2013 |
| **15** | ISP1 (tryptone yeast extract agar) | 0.8836 | +++ | Ahmed et al 2013 |
| **16** | ISP7 (tyrosine agar) | 1.1966 | +++ | Ahmed et al 2013 |
| **17** | Medium without Carbon source | 0.000 | no growth | Contador et al 2015 |
| **18** | Medium without Nitrogen source | 0.000 | no growth | Contador et al 2015 |
| **19** | Medium without Sulfur source | 0.000 | no growth | Contador et al 2015 |
| **20** | Medium without Phosphate source | 0.000 | no growth | Contador et al 2015 |
| **21** | Glucose-Minimal Media Anaerobic | 0.000 | no growth | Ahmed et al 2013, Maldonado et al 2005 |
| **22** | Glucose-Minimal Media Aerobic | growth | growth | Ahmed et al 2013, Maldonado et al 2005 |
| **23** | D-Glucose as carbon source | 1.95 | growth | Contador et al 2015 |
| **24** | D-Mannose as carbon source | 1.95 | growth | Contador et al 2015 |
| **25** | D-Xylose as carbon source | 1.95 | growth | Contador et al 2015 |
| **26** | Glycerol as carbon source | 1.49 | growth | Contador et al 2015 |
| **27** | Lactose as carbon source | 0 | no growth | Contador et al 2015 |
| **28** | Sucrose as carbon source | 1.95 | growth | Contador et al 2015 |
| **29** | D-Fructose as carbon source | 1.95 | growth | Contador et al 2015 |
| **30** | Mannitol as carbon source | 1.95 | growth | Contador et al 2015 |
| **31** | Maltose as carbon source | 1.95 | growth | Contador et al 2015 |
| **32** | Acetate as carbon source | 0.99 | growth | Contador et al 2015 |
| **33** | D-Sorbitol as carbon source | 1.95 | growth | Contador et al 2015 |
| **34** | L-Arabinose as carbon source | 1.95 | growth | Contador et al 2015 |
| **35** | Citrate as carbon source | 1.95 | growth | Contador et al 2015 |
| **36** | L-glutamate as carbon source | 2.48 | growth | Contador et al 2015 |
| **37** | Glycine as carbon source | 0.99 | growth | Contador et al 2015 |
| **38** | Cellobiose as carbon source | 1.95 | growth | Contador et al 2015 |
| **39** | Raffinose as carbon source | 1.95 | growth | Contador et al 2015 |
| **40** | Uracil as carbon source | 0 | no growth | Contador et al 2015 |
| **41** | Uridine as carbon source | 2.07 | growth | Contador et al 2015 |
| **42** | D-Galactose as carbon source | 1.95 | growth | Contador et al 2015 |
| **43** | Lactate as carbon source | 0 | no growth | Contador et al 2015 |
| **44** | Adenosine as carbon source | 3.49 | growth | Contador et al 2015 |
| **45** | Trehalose as carbon source | 1.95 | growth | Contador et al 2015 |
| **46** | Glycine as nitrogen source | 1.95 | growth | Contador et al 2015 |
| **47** | Ammonium as nitrogen source | 1.95 | growth | Contador et al 2015 |
| **48** | Nitrate as nitrogen source | 1.95 | growth | Contador et al 2015 |
| **49** | Urea as nitrogen source | 3.01 | growth | Contador et al 2015 |
| **50** | L-glutamate as nitrogen source | 1.95 | growth | Contador et al 2015 |
| **51** | Nitrite as nitrogen source | 1.95 | growth | Contador et al 2015 |
| **52** | Inosine as nitrogen source | 3.93 | growth | Contador et al 2015 |
| **53** | L-glutamine as nitrogen source | 3.45 | growth | Contador et al 2015 |
| **54** | Adenosine as nitrogen source | 4.01 | growth | Contador et al 2015 |
| **55** | Sulfate as sulfur source | 1.95 | growth | Contador et al 2015 |
| **56** | Sulfite as sulfur source | 1.95 | growth | Contador et al 2015 |
| **57** | L-cysteine as sulfur source | 2.97 | growth | Contador et al 2015 |
| **58** | L-methionine as sulfur source | 2.34 | growth | Contador et al 2015 |
| **59** | Pyrophosphate as phosphorous source | 0 | no growth | Contador et al 2015 |
| **60** | Orthophosphate as phosphorous source | 1.9539 | growth | Contador et al 2015 |
| **61** | β-glycerolphosphate as phosphorous source | 0 | no growth | Contador et al 2015 |
| **62** | Salicin as carbon source | 0 | no growth | Maldonado et al 2005 |
| **63** | L-proline as carbon source | 2.48 | no growth | Maldonado et al 2005 |
| **64** | L-threonine as carbon source | 1.99 | no growth | Maldonado et al 2005 |
| **65** | L-tyrosine as carbon source | 2.01 | no growth | Maldonado et al 2005 |
| **66** | D-galactose as carbon source | 1.95 | growth | Maldonado et al 2005 |
| **67** | L-alanine as nitrogen source | 1.95 | no growth | Maldonado et al 2005 |

PCV = packed cell volumen; SS = synthetic seawater; CM: complex media

+++ = abundant growth in agar medium; ++ = moderate growth in agar medium

**Table S3:** Salinosporamide predicted production rates by iCC926 under different production conditions.

|  |  | **Sal A** | **Sal B** | **Sal A** | **Sal B** |  |
| --- | --- | --- | --- | --- | --- | --- |
|  | **Medium** | **production** | **production** | **production** | **production** | **References** |
|  |  | ***in silico***  **[mmol/gDW h]** | ***in silico***  **[mmol/gDW h]** | ***in vivo***  **[mg/L]** | ***in vivo***  **[mg/L]** |  |
| **68** | Starch-SHY production media | 1.26 | 0.13 | 100 | 13 | Tsueng and Lam, 2009 |
| **69** | Starch-A1 production medium (SS) | 1.26 | 0.13 | 82.2-63.2 |  | Lechner et al, 2011 |
| **70** | Starch-production media plus butyric acid* | 1.01 | 0.22 | 211 | 32.7 | Tsueng and Lam, 2007 |

*experiment with *S. tropica* NPS21184; SS = synthetic seawater; Sal: salinosporamide.

**Table S4:** Salinosporamide predicted production rates of mutant strains by iCC926.

|  |  | **Sal A** | **Sal A** | **Sal B** | **Sal B** |
| --- | --- | --- | --- | --- | --- |
|  | **Strain** | **production** | **production** | **production** | **production** |
|  |  | ***in silico***  **[mmol/gDW h]** | ***in vivo***  ***%*** | ***in silico***  **[mmol/gDW h]** | ***in vivo***  ***%*** |
| **71** | mutant salL- | 0 | n.d | 0.114 | yes |
| **72** | mutant salA- | 0 | n.d | 0 | n.d |
| **73** | mutant salT- | 0.63 | 50 | 0.117 | 91 |
| **74** | mutant salM- | 0.027 | 2.2 | 0.156 | 120 |
| **75** | mutant salH- | 0.048 | 3.8 | 0.091 | 70 |
| **76** | mutant salQ- | 0.315 | 25 | 0.127 | 98 |
| **77** | mutant salG- | 0 | n.d | 0.122 | 94 |
| **78** | mutant Strop_3612- | 1.411 | 112 | 0.067 | 52 |
| **79** | mutant salL- with 5'-ClDA in the media | 2.3 | detected | 0.13 | detected |
| **80** | mutant salL- with 5-CIR in the media | 1.27 | detected | 0.11 | detected |
| **81** | mutant salL- with 5-CIRL in the media | 1.27 | detected | 0.11 | detected |
| **82** | mutant salL- with 5-CIRI in the media | 0.01 | detected | 0.126 | detected |
| **83** | mutant salG- with 5'-ClDA in the media | 0 | n.d | 0.119 | detected |
| **84** | mutant salG- with 5-CIR in the media | 0 | n.d | 0.119 | detected |
| **85** | mutant salG- with 5-CIRL in the media | 0 | n.d | 0.119 | detected |
| **86** | mutant salG- with 5-CIRI in the media | 0 | n.d | 0.119 | detected |

Reference: Eustáquio et al. 2009; Sal: salinosporamide; *in vivo* data: % with respect to wild-type; n.d: not detected

**Table S5:** Sporolides predicted production rates by iCC926 assuming different production ratios.

|  | **Strain** | **Sporolide A** | **Sporolide B** | **Sporolide** | **Ratio** |
| --- | --- | --- | --- | --- | --- |
|  |  | ***in silico***  **[mmol/gDW h]** | ***in silico***  **[mmol/gDW h]** | ***in vivo***  **[ug/L]** | **spo B:spo A** |
| **87** | Wild-type | 0.77 | 0.38 | 100 | 1:2 |
| **88** | Wild-type supplement with tyrosine | 0.906 | 0.453 | 100 |  |
| **89** | Wild-type | 0.578 | 0.578 | 100 | 1:1 |
| **90** | Wild-type supplement with tyrosine | 0.679 | 0.679 | 100 |  |
| **91** | Wild-type | 1.04 | 0.116 | 100 | 1:9 |
| **92** | Wild-type supplement with tyrosine | 1.22 | 0.136 | 100 |  |

Reference: McGlinchey et al 2008b; spo: sporolide

**Table S6:** Sioxanthin predicted production rates by iCC926.

|  | **Strain** | **Sioxanthin** | **Sioxanthin** | **Reference** |
| --- | --- | --- | --- | --- |
|  |  | ***in silico***  **[mmol/gDW h]** | ***in vivo*** |  |
| **93** | Wild-type | 0.221 | detected | Richter et al 2015 |
| **94** | Strop2408mt | 0 | n.d | Richter et al 2015 |
| **95** | Strop3246mt | 0 | n.d | Richter et al 2015 |
| **96** | Strop3247mt | 0 | n.d | Richter et al 2015 |
| **97** | Strop3248mt | 0 | n.d | Richter et al 2015 |

n.d: not detected

**Table S7:** Simulations of *Salinispora arenicola* iSACNH643 model under different growth conditions.

|  | **Medium** | **Growth rate** | **Growth rate** | **References** |
| --- | --- | --- | --- | --- |
|  |  | ***in silico*** | **in vivo** |  |
|  |  | **1/h** |  |  |
| **1** | Medium without Carbon source | 0.000 | no growth | Contador et al 2015 |
| **2** | Medium without Nitrogen source | 0.000 | no growth | Contador et al 2015 |
| **3** | Medium without Sulfur source | 0.000 | no growth | Contador et al 2015 |
| **4** | Medium without Phosphate source | 0.000 | no growth | Contador et al 2015 |
| **5** | Glucose-Minimal Media Anaerobic | 0.000 | no growth | Ahmed et al 2013, Maldonado et al 2005 |
| **6** | Glucose-Minimal Media Aerobic | growth | growth | Ahmed et al 2013, Maldonado et al 2005 |
| **7** | Starch-A1 medium | 1.9698 | growth | This work |
| **8** | ISP2 (glucose-malt-yeast extract agar) | 1.0687 | +++ | Ahmed et al 2013 |
| **9** | ISP5 (glycerol-asparagine agar) | 0.9611 | +++ | Ahmed et al 2013 |
| **10** | ISP4 (inorganic salts-starch agar) | 0.5248 | ++ | Ahmed et al 2013 |
| **11** | ISP3 (oatmeal agar) | 0.6736 | +++ | Ahmed et al 2013 |
| **12** | ISP6 (peptone-yeast extract-iron agar) | 0.8502 | +++ | Ahmed et al 2013 |
| **13** | ISP1 (tryptone yeast extract agar) | 1.7004 | +++ | Ahmed et al 2013 |
| **14** | ISP7 (tyrosine agar) | 0.8822 | +++ | Ahmed et al 2013 |
| **15** | DMM | 0.1749 | growth | This work |
| **16** | Salicin as carbon source | 2.0839 | growth | Maldonado et al 2005 |
| **17** | L-proline as carbon source | 2.5271 | growth | Maldonado et al 2005 |
| **18** | L-threonine as carbon source | 2.3325 | growth | Maldonado et al 2005 |
| **19** | L-tyrosine as carbon source | 2.3958 | growth | Maldonado et al 2005 |
| **20** | D-galactose as carbon source | 0 | no growth | Maldonado et al 2005 |
| **21** | L-alanine as nitrogen source | 2.0839 | growth | Ahmed et al 2013 |
| **22** | L-glutamate as nitrogen source | 2.0839 | no growth | Ahmed et al 2013 |
| **23** | Sioxanthin production | 0.1941 mmol/gDW h | detected | Maldonado et al 2005; this work |

+++ = abundant growth in agar medium; ++ = moderate growth in agar medium

**Table S8:** Simulations of *Salinispora pacifica* iSPCNR114 model under different growth conditions.

|  | **Medium** | **Growth rate** | **Growth rate** | **References** |
| --- | --- | --- | --- | --- |
|  |  | ***in silico*** | **in vivo** |  |
|  |  | **1/h** |  |  |
| **1** | Medium without Carbon source | 0.000 | no growth | Contador et al 2015 |
| **2** | Medium without Nitrogen source | 0.000 | no growth | Contador et al 2015 |
| **3** | Medium without Sulfur source | 0.000 | no growth | Contador et al 2015 |
| **4** | Medium without Phosphate source | 0.000 | no growth | Contador et al 2015 |
| **5** | Glucose-Minimal Media Anaerobic | 0.000 | no growth | Ahmed et al 2013, Maldonado et al 2005 |
| **6** | Glucose-Minimal Media Aerobic | growth | growth | Ahmed et al 2013, Maldonado et al 2005 |
| **7** | Starch-A1 medium | 1.3673 | growth | Ahmed et al 2013 |
| **8** | ISP2 (glucose-malt-yeast extract agar) | 0.9013 | +++ | Ahmed et al 2013 |
| **9** | ISP5 (glycerol-asparagine agar) | 0.8426 | +++ | Ahmed et al 2013 |
| **10** | ISP4 (inorganic salts-starch agar) | 0.9306 | ++ | Ahmed et al 2013 |
| **11** | ISP3 (oatmeal agar) | 0.5690 | +++ | Ahmed et al 2013 |
| **12** | ISP6 (peptone-yeast extract-iron agar) | 0.148 | +++ | Ahmed et al 2013 |
| **13** | ISP1 (tryptone yeast extract agar) | 1.48 | +++ | Ahmed et al 2013 |
| **14** | ISP7 (tyrosine agar) | 0.9853 | +++ | Ahmed et al 2013 |
| **15** | DMM | 0.1469 | growth | This work |
| **16** | L-alanine as nitrogen source | 1.8612 | no growth | Ahmed et al 2013 |
| **17** | L-glutamate as nitrogen source | 18612 | growth | Ahmed et al 2013 |
| **18** | Sioxanthin production | 0.1395 mmol/gDW h | detected | Ahmed et al 2013; this work |
| **19** | Lomaiviticin A production | 0.3633 mmol/gDW h | detected | Duncan et al 2015 |

+++ = abundant growth in agar medium; ++ = moderate growth in agar medium

**
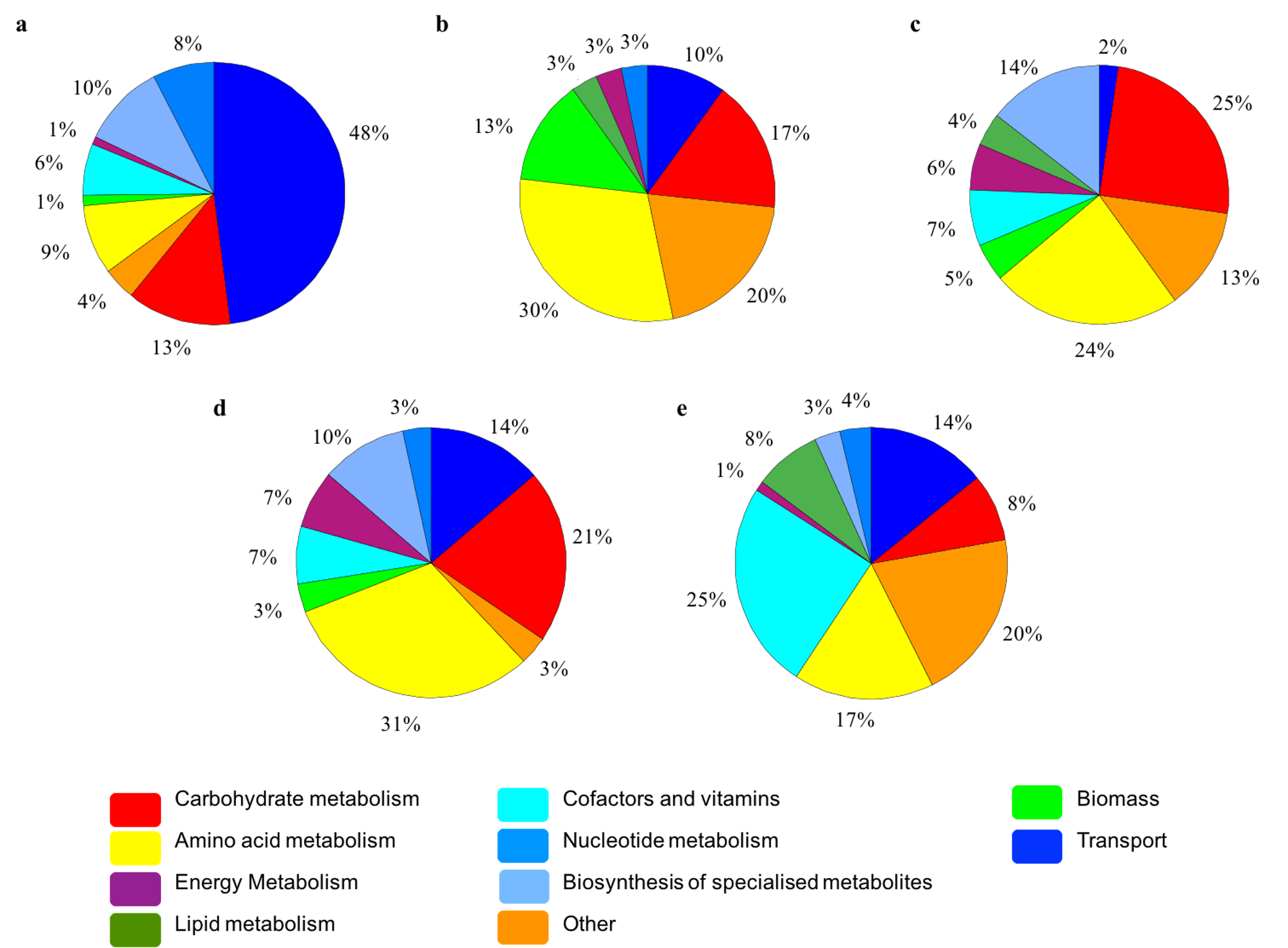
**

**Figure S5** Distribution of unique sets of reactions by subsystems. (a) ST-CNB440; (b) SA-CNH643; (c) SP-CNR114; (d) ST-CNB440/SA-CNH643; (e) ST-CNB440/SA-CNH643. Sets with 20 or more reactions were represented.

**References**

1. Ahmed L, Jensen PR, Freel KC, Brown R, Jones AL, Kim BY, Goodfellow M (2013). Salinispora pacifica sp. nov., an actinomycete from marine sediments. Antonie van Leeuwenhoek, Int. J. Gen. Mol. Microbiol. 103, 1069–1078.
2. Alam MT, Merlo ME, Consortium TS, Hodgson DA, Wellington EMH, Takano E, Breitling R (2010). Metabolic modeling and analysis of the metabolic switch in Streptomyces coelicolor. BMC Genomics 11, 202.
3. Contador CA, Rodríguez V, Andrews BA, Asenjo JA. Genome-scale reconstruction of Salinispora tropica CNB-440 metabolism to study strain-specific adaptation. Antonie van Leeuwenhoek, Int. J. Gen. Mol. Microbiol. 2015;108:1075–90.
4. Eustáquio AS, McGlinchey RP, Liu Y, Hazzard C, Beer LL, Florova G, Alhamadsheh MM, Lechner A, Kale AJ, Kobayashi Y, Reynolds KA, Moore BS (2009). Biosynthesis of the salinosporamide A polyketide synthase substrate chloroethylmalonyl-coenzyme A from S-adenosyl-L-methionine. Proc. Natl. Acad. Sci. U. S. A. 106, 12295–12300.
5. Lechner A, Eustáquio AS, Gulder TAM, Hafner M, Moore BS (2011). Selective overproduction of the proteasome inhibitor salinosporamide A via precursor pathway regulation. Chem. Biol. 18, 1527–1536.
6. Maldonado LA, Fenical W, Jensen PR, Kauffman CA, Mincer TJ, Ward AC, Bull AT, Goodfellow M (2005). Salinispora arenicola gen. nov., sp. nov. and Salinispora tropica sp. nov., obligate marine actinomycetes belonging to the family Micromonosporaceae. Int. J. Syst. Evol. Microbiol. 55, 1759–1766
7. McGlinchey RP, Nett M, Moore BS (2008b). Unraveling the biosynthesis of the sporolide cyclohexenone building block. J. Am. Chem. Soc. 130, 2406–2407.
8. Tsueng G, McArthur KA, Potts BCM, Lam KS (2007). Unique butyric acid incorporation patterns for salinosporamides A and B reveal distinct biosynthetic origins. Appl. Microbiol. Biotechnol. 75, 999–1005.
9. Tsueng G, Lam KS (2008b). Growth of Salinispora tropica strains CNB440, CNB476, and NPS21184 in nonsaline, low-sodium media. Appl. Microbiol. Biotechnol. 80, 873–880.
10. Tsueng G, Teisan S, Lam KS (2008). Defined salt formulations for the growth of Salinispora tropica strain NPS21184 and the production of salinosporamide A (NPI-0052) and related analogs. Appl. Microbiol. Biotechnol. 78, 827–832.
11. Tsueng G, Lam KS (2009). Effect of cobalt and vitamin B12 on the production of salinosporamides by Salinispora tropica. J. Antibiot. 62, 213–216.
12. Tsueng G, Lam KS (2010). A preliminary investigation on the growth requirement for monovalent cations, divalent cations and medium ionic strength of marine actinomycete Salinispora. Appl. Microbiol. Biotechnol. 86, 1525–1534.
13. Richter TKS, Hughes CC, Moore BS. Sioxanthin, a novel glycosylated carotenoid, reveals an unusual subclustered biosynthetic pathway. Environ. Microbiol. 2015;17:2158–71.
14. Duncan KR, Crüsemann M, Lechner A, Sarkar A, Li J, Ziemert N, et al. Molecular Networking and Pattern-Based Genome Mining Improves Discovery of Biosynthetic Gene Clusters and their Products from Salinispora Species. Chem. Biol. 2015;460–71.
